# Supplementary material for: The interactome of intact mitochondria by cross-linking mass spectrometry provides evidence for coexisting respiratory supercomplexes
Source: Mol Cell Proteomics. 2017 Dec 8;17(2):216–32. doi: 10.1074/mcp.RA117.000470 (PMC5795388; doi:10.1074/mcp.RA117.000470)
Supplement: Supplemental Data [file supp_RA117.000470_133922_0_supp_23978_fzffwf.zip › spectra_annotation/mito_DR_spectra_annotation/377-1-4-1-7-1.pdf]

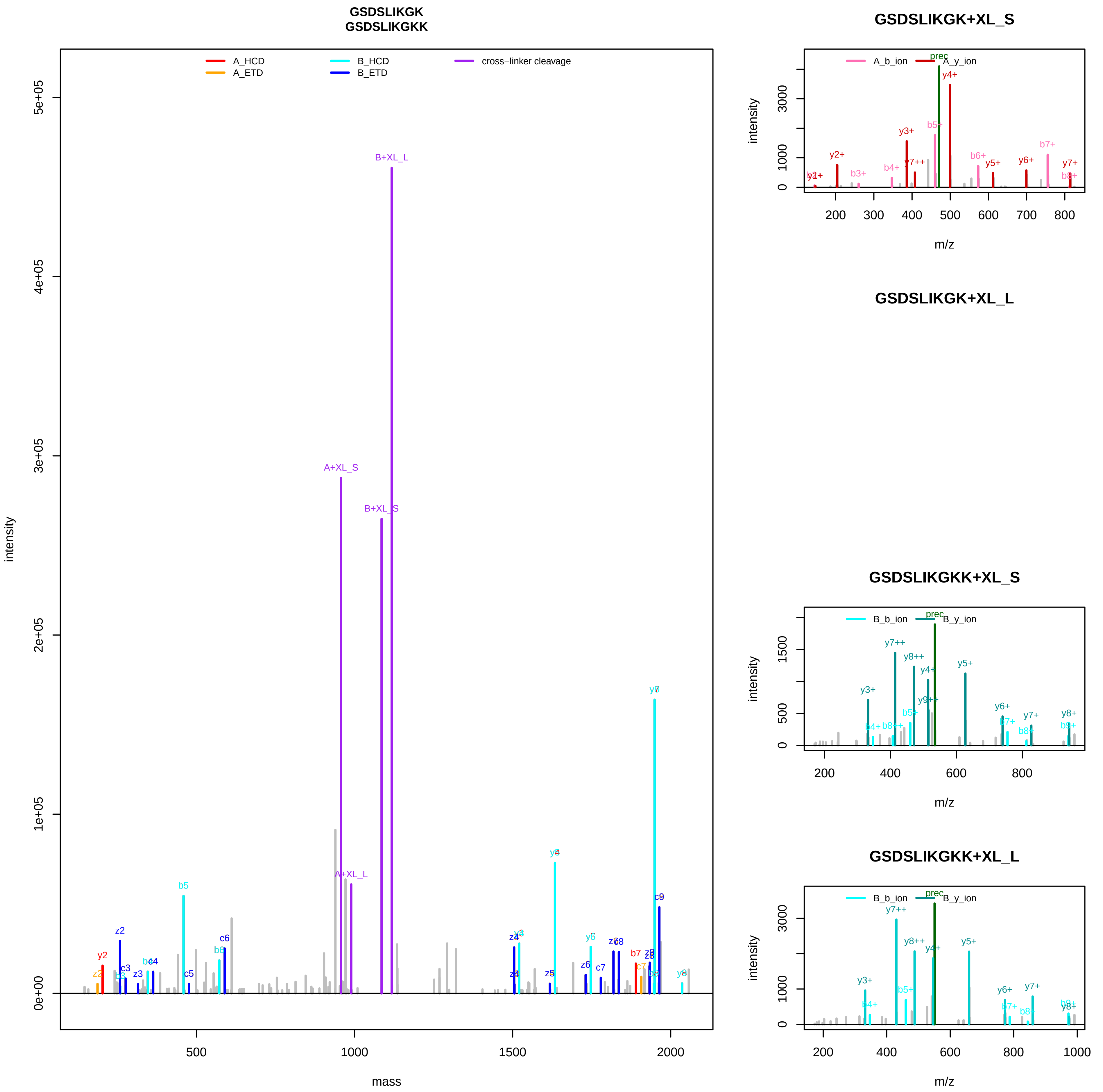
GSDSLIKGK
GSDSLIKGKK
A_HCD
A_ETD
B_HCD
B_ETD
cross−linker cleavage
B+XL_L
A+XL_S
B+XL_S
A+XL_L
intensity
mass
5e+05
4e+05
3e+05
2e+05
1e+05
0e+00
500
1000
1500
2000
y2
z2
c3
z3
c4
b5
c5
c6
b6
z4
y3
y4
z5
z6
y5
c7
z7
c8
b7
c7
z8
y7
c9
y8
GSDSLIKGK+XL_S
A_b_ion
A_y_ion
prec
y1+
y2+
b3+
b4+
y3+
y7++
b5+
y4+
b6+
y5+
y6+
b7+
y7+
b8+
intensity
m/z
GSDSLIKGK+XL_L
GSDSLIKGKK+XL_S
B_b_ion
B_y_ion
y3+
b4+
b8++
y7++
y8++
y4+
b5+
y9++
y5+
y6+
b7+
y7+
b8+
y8+
b9+
GSDSLIKGKK+XL_L
